# Three measures of internet use, social media use and video game playing as predictors of insomnia during the pandemic among students

**DOI:** 10.1038/s41598-024-53351-2

**Published:** 2024-02-08

**Authors:** Špela Selak, Andrej Šorgo, Nuša Crnkovič, Branko Gabrovec, Katarina Cesar, Mark Žmavc

**Affiliations:** 1 National Institute of Public Health, Ljubljana, Slovenia; 2 Faculty of Natural Sciences and Mathematics, University of Maribor, Maribor, Slovenia

**Keywords:** Psychology, Risk factors, Signs and symptoms

## Abstract

Existing research indicates that the relationship between digital technology use and insomnia can largely depend on which digital technology measure and which insomnia measure is considered. Data on 4261 Slovenian tertiary students was gathered through an online survey in February 2021, which included measures of depression and insomnia symptoms, as well as measures of internet, social media and video game use divided into three measurement levels (use, duration of use, addictive use). Regression analysis revealed an apparent effect of measurement level, where addictive use measures consistently outperformed other technology use measures in predicting insomnia. Regardless of measurement level, social media use seems to produce more risk for insomnia, compared to playing video games or general internet use for leisure purposes. Importantly, a single measure of depression symptoms explained more variance in insomnia than the nine measures of digital technology use combined, meaning that the effect of digital technology on sleep should not be overstated. Most of the effect of social media use on insomnia may in fact be explained by understanding users' depression symptoms. In case of gaming, a larger part of its effect on insomnia is independent of depression symptoms.

## Introduction

The COVID-19 pandemic has had a significant impact on the mental health of the general population^1,2^, including an increase in sleep disturbances such as insomnia^3^. The International classification of sleep disorders^4^, the Diagnostic and statistical manual of mental disorders^5^ (DSM-5) and the International classification of diseases^6^ (ICD-11) include similar descriptions of insomnia; a sleep disorder characterized by persistent difficulties with initiating and/or maintaining sleep despite adequate opportunity for sleep, which result in (clinically) significant daytime impairment. Zitting and colleagues^7^ report that during the first half of 2020, Google search queries for “insomnia” significantly increased worldwide compared to the same periods in previous years and were as much as 58% more frequent (in the United States). Over the course of the pandemic, it became apparent that Slovenian university students may be particularly vulnerable to mental health decline, due to the enduring closure of faculties and student dormitories, making many students return to live with their parents. Studies on the trajectory of insomnia symptoms during the COVID-19 pandemic confirmed the increased prevalence and worsening of insomnia symptoms among university students^8,9^.

### Pandemic-related effects on sleep

One consequence of government-imposed restrictions to prevent the spread of COVID-19 was the emergence of a specific set of stressors that could have led to disrupted sleep patterns, including fear of infection, uncertainty about the future, home confinement, social isolation, financial strain, etc. A study by Meaklim and colleagues^10^ revealed that adults who have begun experiencing insomnia after the start of the pandemic reported significantly higher levels of perceived stress during the pandemic than the no-insomnia group and pre-existing insomnia group, whereas 77% of those individuals believe that the factors surrounding the pandemic triggered their insomnia symptoms.

In addition, pandemic-related changes in lifestyle, such as decreased physical activity, increased screen time, and irregular sleep–wake schedules have been linked to an exacerbation of sleep difficulties. Physical inactivity during the first wave of the COVID-19 pandemic was found to predict insomnia symptoms in Spanish men and women^11^, while a more recent study^12^ showed that the irregularity of sleep–wake schedules and exposure to LED light (screens) during the evening significantly predicted the likelihood of experiencing insomnia symptoms as well.

### The effect of digital technologies on sleep

The effect of using digital technologies on sleep quality or quantity has been a relatively popular study topic in recent times. One established explanation of how screen time in the evening can influence sleep argues that blue light exposure, emitted by most commercially available electronic devices, suppresses the secretion of melatonin, a hormone that directly regulates the circadian rhythm^13^. Even so, the relationship between technology and sleep should not be reduced to the effects of light exposure. Using digital technologies late in the evening may also lead to increased cognitive arousal, which is recognized as a prominent factor of insomnia^4,5^. This implies the crucial role of the type of digital content a person is interacting with before going to bed. For example, listening to calming music online in the evening can have quite opposite effects on sleep compared to playing competitive video games. Hence, the effects of various digital technologies on sleep should not be generalized, but should instead be understood with respect to the content and the context of digital technology use.

### Video games and sleep

Briefly looking at the relevant literature in the field of video games, a systematic review by Pelletier and colleagues^14^ identified five studies, in which either weak associations or no associations were found between gaming time and lack of sleep or level of fatigue. Similarly, Altintas and colleagues^15^ have found that rather than duration, the intensity of gaming was the more salient predictor of poor sleep quality, which lends support to the cognitive arousal hypothesis (see above). This notion was experimentally tested in a study^16^, which observed that pre-sleep video game playing increased sleep onset latency and decreased subjective sleepiness, though only slightly. Pre-sleep video game playing was also associated with increased cognitive alertness, while having no effect on a player’s physiological arousal in the same study. Finally, Fazeli and colleagues^17^ showed a direct effect of internet gaming disorder symptoms on insomnia scores during the COVID-19 outbreak.

### Social media and sleep

In the research on the effects of social media on sleep, many measures of social media use have also been utilized. Social media use before bed seems to have some detrimental effects on sleep, e.g. increase in sleep latency (but no effect on sleep continuity^18^) and insomnia scores^19^. The duration of social media use was also found to be associated with sleep problems^20,21^, while frequency of social media use was an important predictor of insomnia in two studies^19,21^, but had no relation to insomnia in another study^20^. Another common measure, namely addictive use of social media, was associated with an increase in insomnia symptoms after 3 months in a longitudinal study^22^.

### Rationale of the study and hypotheses

A short review of studies within these two research areas (i.e., the effect of social media/video games on sleep) makes it obvious that the conclusions regarding the effects largely depend on which measure of digital technology use and which measure of sleep quality or quantity is considered. Thus, the present study addresses the issue of conflicting evidence in this area through analyzing the differences in the predictive power of various digital technology use measures. Firstly, we focus on how well insomnia is predicted by the measures of use among different technology types, i.e. internet use, social media use and video game use. We expected to find a significant effect of digital technology type. Secondly, the measures of digital technology use were separated into three measurement levels; use (yes/no), duration of use, and addictive use. We expected to find a significant effect of measurement level on the capacity of variables to predict insomnia. We expected the third level of measurement within all three technology categories, i.e. addictive use, would function as the best predictor of insomnia, on the grounds that these measures should capture an individual’s lack of self-control regarding technology use and the consequent likelihood of sacrificing sleep quality or quantity in favor of technology use. Lastly, in line with the reasoning above, the effect of these measures was studied separately in two insomnia measures; insomnia symptoms (total score) and clinical insomnia (absence/presence).

## Methodology

### Participants

The target sample were the tertiary students in Slovenia, including both university students and students of any other institutions offering post-secondary education, who were enrolled in the study year of 2020/2021. Due to the use of Slovenian language in the survey, all participants who completed the survey were presumably fluent in Slovene. According to the Slovenian statistical office^23^, there were 82,694 full-time tertiary students in Slovenia in 2020/2021, of which 42.4% were male, and 57.6% were female. Out of 5999 students who submitted the survey, 71.0% (N = 4261—5.2% of the population) provided complete responses to the questions relevant in the present study and thus formed the final sample. Taking the gender ratio in the population into account, female students were more likely to respond to the survey; 73.0% of participants identified as women (n = 3109), 26.3% as men (n = 1121) and 0.7% as other (n = 30). The students were 22.9 years old on average (SD = 3.2, Min = 18, Max = 57), while most (97.6%) were younger than 30. More than half of the sample reported they were not in a relationship (55.0%, n = 2345), and only 2.8% (n = 120) reported to be living alone at the time.

### Procedure

Firstly, an ethical approval to conduct the study was obtained from the National Medical Ethics Committee of the Republic of Slovenia (NMEC), Ministry of Health (No. 0120-48/2021/3). The creation of the questionnaire, recruitment of participants, as well as gathering and processing of data—i.e. the entire methodology, was carried out in accordance with the relevant guidelines and regulations. The data used in this paper was gathered through a larger cross-sectional study, the focus of which were various aspects of mental health and wellbeing in Slovenian tertiary students during the COVID-19 pandemic. All Slovenian universities, private faculties and student organizations were invitated to contribute to the study by sharing the online survey link with all their students via e-mail, as well as through their official webpages and social media accounts. Participants were informed about the key aspects of the study, their right to withdraw from it at any time without any consequences whatsoever; they were assured that their data would remain anonymous, and that the gathered data will be processed in accordance with the European Union and Slovenian legislation. Following this, all participants were asked to provide an informed consent to participate in the study. As a result, all participants gave an informed consent before answering any of the questionnaire questions in the present study. Data collection was performed with the help of a web-based survey between 9 February and 8 March 2021.

### Instruments and measures

#### The Insomnia Severity Index

The Insomnia Severity Index (ISI)^24^ is a brief screening tool for insomnia with seven items asking participants to rate the nature and symptoms of their sleep problems using a 5-point Likert scale (e.g., 0—Not at all, 1—A little, 2—Some, 3—Quite a bit, 4—A lot) with a total score ranging from 0 to 28 points^24^. The sum of total scores is interpreted as follows: insomnia is not clinically significant (0–7 points); insomnia is below the normal threshold (8–14 points); clinical insomnia (moderate expression) (15–21 points); clinical insomnia (severe expression) (22–28 points). Statistical analysis utilizes two insomnia measures based on ISI, the total insomnia score and the presence of clinical insomnia (> 15 points).

#### Internet Disorder Scale

The Internet Disorder Scale (IDS-15)^25^ is a 15-item measure assessing the severity of Internet addiction among internet users, and is based on the DSM-5 framework for Internet gaming disorder^5^. The set of items focuses on users' online leisure activities (those not relating to work or school) within the last 12 months across any internet-enabled devices. These items are grouped into four distinct domains: Escapism and Dysfunctional Emotional Coping (EDEC); Withdrawal Symptoms (WS); Impairments and Dysfunctional Self-Regulation (IDSR); and Dysfunctional Internet-related Self-Control (DISC). Participants are required to rate their responses on a Likert Scale, ranging from 1 (“Strongly disagree”) to 5 (“Strongly agree”), with higher scores indicating a higher risk or severity of internet addiction. The total scores can range between 15 and 75 points. The scale was adapted to the Slovenian language and reported adequate psychometric properties of the Slovenian IDS-15^26^. The internal consistency of the scale in the present study was α = 0.89.

#### Bergen Social Media Addiction Scale

The Bergen Social Media Addiction Scale (BSMAS)^27^ is a set of six items that assess a person's relationship with the social media. The instructions define the social media as “Facebook, Twitter, Instagram, and the like”. Responses are provided on a 5-point Likert scale, with options ranging from “Very rarely” to “Very often”, resulting in a score between 6 and 30. A higher score indicates a greater likelihood of social media addiction. The scale was adapted to the Slovenian language and reported adequate psychometric properties of the Slovenian BSMAS^28^. The internal consistency of the scale in the present study was α = 0.87.

#### Internet Gaming Disorder Scale–Short-Form

To assess the severity of gaming addiction and its negative impacts over the past year, we used the Internet Gaming Disorder Scale–Short-Form (IGDS9-SF)^29^, a nine-item psychometric tool to assess Internet gaming disorder according to the DSM-5 criteria^5^. Participants are asked to rate items such as “Do you struggle to control or stop your gaming activity?” using a 5-point Likert scale ranging from “Never” to “Very often”. Total scores range from 9 to 45, with higher scores indicating more severe Internet gaming disorder. The IGDS9-SF is a validated tool for evaluating Internet gaming disorder across different cultures^30^. Pontes and colleagues^31^ adapted and validated a Slovenian version of the tool in a study among Slovenian youth. The internal consistency of the scale in the current study was α = 0.88.

#### The Patient Health Questionnaire

The Patient Health Questionnaire^32^ (PHQ-9) was used to evaluate depression symptoms over the past 14 days. This screening tool is widely used to assess depression in studies related to the COVID-19 pandemic. The PHQ-9 consists of 9 items, based on DSM-V diagnostic criteria, which evaluate depressive symptoms on a 4-point Likert scale, with scores ranging from 0 (“Not at all”) to 3 (“Nearly every day”). The total score can range from 0 to 27, with higher scores indicating a greater presence of depressive symptoms, categorized as minimal (1–4), mild (5–9), moderate (10–14), moderately severe (15–19), and severe (20–27)^33^. A cut-off point of 10 or higher is commonly used to identify participants with depressive symptoms. The internal consistency of the PHQ-9 in the current study was α = 0.91.

#### Other measures

Among other relevant measures of the survey were the questions regarding students’ use of internet, social media and video games; i.e., whether they use these technologies, and if so, how much time per day they spend engaging with each digital technology type. These questions prompted the participants to report the use of these technologies in their free time (excluding the use of internet, social media or video games for the purposes of their study or work). Many demographic questions were also included in the survey, but only gender was included in the analysis of this paper.

### Statistical analysis

The statistical methods used consisted of the following: (i) frequencies and descriptive statistics of all included variables; (ii) *t*-test between male and female students after testing assumptions; (iii) assumptions testing for performing a multiple linear regression: independence of observations, linearity of relationships, normality of residuals, no extreme outliers, absence of multicollinearity and homoscedasticity; (iv) assumptions testing for performing a binary logistic regression: independence of observations, absence of multicollinearity, no extreme outliers, and linearity of the relationship between the predictor and the log odds of the criterion variable; (v) hierarchical multiple linear regression to evaluate the contribution of various predictor variables towards insomnia scores, and to obtain and compare the variance explained (R^2^) by the four groups of factors; and (vi) binary logistic regression to evaluate the contribution of various predictor variables towards the presence of clinical insomnia.

We did not find any major violations of *t*-test, linear regression and binary logistic regression assumptions. The two regression methods included four predictive models of insomnia each. The first predictive model included measures of whether a certain technology is used, the second model included the variables describing duration of use, the third model included addictive use variables and the last model included depression symptoms as a predictor of insomnia. In the case of multiple linear regression, the predictive variables in subsequent models were added to the previous variables, while in the case of binary logistic regression, the predictive variables in subsequent models replaced previous variables. All statistical analysis was carried out with SPSS.

### Ethics approval

Ethical approval to conduct the study was obtained from the National Medical Ethics Committee of the Republic of Slovenia (NMEC), Ministry of Health (No. 0120-48/2021/3).

## Results

### Descriptive statistics

Among the students in the sample, 99.8% reported to use the internet in their free time, 97.7% were social media users and 43.3% were video game players. Descriptive statistics of other key variables and used scales are shown in Table 1. Students were spending 4.71 h per day for various leisure activities on the internet. More than half of this time was spent on social media (2.74 h). Lastly, gaming was the least time-consuming activity for the average student (less than one hour daily).

**Table 1:** Descriptive statistics of key variables and scales (N = 4261).

	Min	Max	M	Me	SD
Internet hours (daily)	0	19	4.71	4.00	2.87
Social media hours (daily)	0	19	2.74	2.29	2.05
Gaming hours (daily)	0	13	0.80	0.00	1.48
IDS-15	15	74	39.52	39.00	10.76
BSMAS	6	30	13.64	13.00	5.72
IGDS9-SF	9	45	11.26	9.00	4.77
PHQ-9	0	27	11.26	11.00	7.19
ISI	0	28	9.71	9.00	6.57

Looking at students’ self-assessment of their insomnia symptoms more closely (Table 2), it is remarkable that almost every fourth student (24.5%) could be classified into one of the two clinical categories, having either clinical insomnia (moderate expression) or clinical insomnia (severe expression). Insomnia severity scores are higher among female students compared to their male peers (*t*(4228) = 3.771, *p* < 0.001, Cohen’s d = 0.13).

**Table 2:** Proportions of students in each of the four insomnia severity categories.

Insomnia category	n	%
None	1790	42.0
Mild	1429	33.5
Clinical—moderate	848	19.9
Clinical—severe	194	4.6
Total	4261	100.0

### Predictors of insomnia symptoms

Table 3 presents the summary of the four regression models, where different types of measures of digital technology use (internet use, social media use and video game use) and one measure of depression were used to predict students’ insomnia scores. Comparing the variance in insomnia scores explained by each group of predictors (R^2^), it is evident that whether or not a student uses a certain digital technology in their free time explains only 0.3% of variance in insomnia scores (Model 1). Looking at standardized regression coefficients (β) in Table 4, we find that internet use in spare time (this does not refer to the duration of use, but whether or not someone uses the internet in their spare time) has next to no predictive value for insomnia scores (since most students are internet users anyway). On the other hand, social media and video game users did obtain higher insomnia scores than their peers who do not use these technologies, although the differences between them were rather small, judging by the low beta coefficients (β = 0.047 and β = 0.037).

**Table 3:** Proportions of explained variance in insomnia scores for each of the four predictive models.

Model	R	R^2^	R^2^ change	F change	df1	df2	p
1	0.059	0.003	0.003	4.97	3	4257	0.002
2	0.199	0.040	0.036	53.59	3	4254	0.000
3	0.364	0.133	0.093	151.46	3	4251	0.000
4	0.670	0.449	0.317	2446.24	1	4250	0.000

**Table 4:** Standardized regression coefficients of insomnia predictors in the four models and their statistical significance.

Model	Variable	Standardized coefficients			Correlations	
		β	t	p	Zero-order	Semi-partial
1	(Constant)		2.727	0.006		
	**Internet use**	− 0.004	− 0.230	0.818	− 0.003	− 0.004
	**Social media use**	0.047	3.097	0.002	0.046	0.047
	**Video game use**	0.037	2.401	0.016	0.035	0.037
2	(Constant)		3.064	0.002		
	Internet use	− 0.013	− 0.868	0.385	− 0.003	− 0.013
	Social media use	0.027	1.753	0.080	0.046	0.026
	Video game use	0.033	1.711	0.087	0.035	0.026
	**Internet hours (daily)**	0.084	4.702	0.000	0.149	0.071
	**Social media hours (daily)**	0.139	8.122	0.000	0.179	0.122
	**Gaming hours (daily)**	− 0.003	− 0.154	0.878	0.038	− 0.002
3	(Constant)		1.374	0.170		
	Internet use	− 0.021	− 1.433	0.152	− 0.003	− 0.020
	Social media use	0.009	0.623	0.533	0.046	0.009
	Video game use	0.013	0.671	0.502	0.035	0.010
	Internet hours (daily)	0.048	2.743	0.006	0.149	0.039
	Social media hours (daily)	0.033	1.866	0.062	0.179	0.027
	Gaming hours (daily)	− 0.043	− 2.004	0.045	0.038	− 0.029
	**IA symptoms**	0.155	7.287	0.000	0.323	0.104
	**SMA symptoms**	0.169	7.786	0.000	0.307	0.111
	**GD symptoms**	0.119	6.016	0.000	0.154	0.086
4	(Constant)		0.809	0.418		
	Internet use	− 0.001	− 0.055	0.956	− 0.003	− 0.001
	Social media use	0.000	0.034	0.973	0.046	0.000
	Video game use	0.003	0.230	0.818	0.035	0.003
	Internet hours (daily)	0.002	0.116	0.908	0.149	0.001
	Social media hours (daily)	0.007	0.480	0.631	0.179	0.005
	Gaming hours (daily)	− 0.025	− 1.447	0.148	0.038	− 0.016
	IA symptoms	0.000	0.011	0.991	0.323	0.000
	SMA symptoms	0.041	2.359	0.018	0.307	0.027
	GD symptoms	0.065	4.111	0.000	0.154	0.047
	**Depression symptoms**	0.639	49.460	0.000	0.667	0.563

In each model, the newly added predictors are highlighted in bold.

Referring back to Table 3, we see that the duration of digital technology use in students’ spare time only adds another few percentage points to the proportion of explained variance in insomnia scores, adding up to 4% in total (Model 2). Looking at the regression coefficients and their significance in Table 4 we find that *Internet daily hours* (β = 0.084) and *Social media daily hours* (β = 0.139) significantly predict insomnia scores, while *Gaming daily hours* (β = − 0.003) do not.

Model 3, which additionally contains the information about the extent to which students experience addiction-like symptoms in relation to each digital technology, explains a further 9% of variance in insomnia scores, adding up to 13% of variance in total (Table 3). Each of the three newly included variables, namely *IA* (Internet Addiction) *symptoms*, *SMA* (Social Media Addiction) *symptoms* and *GD* (Gaming Disorder) *symptoms*, are significant predictors of insomnia and are by far the three strongest predictors of insomnia among the digital technology use variables (β = 0.155, β = 0.169, β = 0.119). It is important to observe that measures of social media use seem to be stronger predictors of insomnia scores compared to measures of internet use and measures of video game use, regardless of the measurement level. *Social media use, Social media hours (daily)* and *SMA* *symptoms* yield higher beta coefficients compared to the respective measures of the other two technology types (see the regression coefficients in Table 4).

The final predictive model (i.e. Model 4), which additionally includes students’ self-reported depression symptoms, markedly improves the variance explained in insomnia scores. Introducing a single new variable to the nine existing predictors adds 32 percentage points to the variance explained by the model, adding up to 45% in total (Table 3). Table 4 shows that experiencing depression symptoms is very strongly associated with experiencing insomnia (β = 0.64). Taking depression scores into account, only two digital technology use variables remain significant (though still relatively weak) predictors of insomnia, namely *SMA symptoms* and *GD symptoms*. Notably, *IA symptoms* completely lose their independent predictive power after accounting for students’ depression scores (β = 0.000), while *GD symptoms* become a better predictor than *SMA symptoms*.

### Predictors of clinical insomnia

Table 5 shows all of the variables as predictors of clinical insomnia (a binary variable corresponding to either absence or presence of clinical insomnia; this does not refer to a clinical diagnosis of insomnia but to a high score on the Insomnia Severity Index tool) in four separate predictive models. Characteristics of Model 5 variables indicate that whether or not someone is an internet user or a video game player in their spare time does not significantly predict clinical insomnia. On the other hand, the use of social media in spare time (reported by most students) does significantly predict clinical insomnia. Nevertheless, according to the Nagelkerke R^2^ value, the three variables only explain 0.5% of variance in clinical insomnia.

**Table 5:** Binary logistic regression coefficients and odds ratios for predicting clinical insomnia.

Model	Variable	B	S.E.	Wald	Sig	Exp(B)	95% C.I. for EXP(B)	
							Lower	Upper
5	Internet use	− 0.89	0.67	1.75	0.186	0.411	0.110	1.533
	Social media use	0.96	0.31	9.69	0.002	2.612	1.427	4.781
	Video game use	0.01	0.07	0.03	0.870	1.011	0.883	1.159
	Constant	− 1.19	0.74	2.58	0.108	0.306		
6	Internet hours (daily)	0.05	0.01	14.08	0.000	1.053	1.025	1.082
	Social media hours (daily)	0.11	0.02	37.32	0.000	1.116	1.077	1.156
	Gaming hours (daily)	0.00	0.02	0.00	0.959	0.999	0.952	1.048
	Constant	− 1.69	0.07	532.19	0.000	0.184		
7	IA symptoms	0.02	0.00	12.94	0.000	1.017	1.008	1.027
	SMA symptoms	0.07	0.01	72.38	0.000	1.075	1.057	1.093
	GD symptoms	0.03	0.01	21.32	0.000	1.034	1.020	1.049
	Constant	− 3.26	0.16	436.59	0.000	0.038		
8	Depression symptoms	0.20	0.01	885.31	0.000	1.219	1.203	1.235
	Constant	− 3.81	0.11	1218.02	0.000	0.022		

Expectedly, the variables representing the duration of digital technology use (Model 6) perform better as the predictors of clinical insomnia, as they account for 3.1% of its variance. Interestingly, *Internet hours (daily)* and *Social media hours (daily)* turn out to be significant predictors, while *Gaming hours (daily)* are not. Looking at the odds ratios (Exp(B) in Table 5) an additional hour of internet use per day (in spare time) is associated with a 5% increased likelihood of clinical insomnia, while an additional hour of social media per day (in spare time) is associated with a 12% increased likelihood.

The variables representing the addictive use of digital technologies (Model 7) explain 9.5% of variance in clinical insomnia. In this case, all three variables seem to significantly predict the likelihood of clinical insomnia. The presented odds ratios reveal that a one-point increase on the Internet disorder scale (scores ranging from 15 to 75) is associated with a 1.7% increased likelihood of clinical insomnia, a one-point increase on the Bergen social media addiction scale (ranging from 6 to 30) is associated with a 7.5% increased likelihood of clinical insomnia, and a one-point increase on the Internet gaming disorder scale (ranging from 9 to 45) is associated with a 3.4% increased likelihood of clinical insomnia.

Lastly, Model 8 (Table 5), which contains depression symptoms as the only predictive variable, explains 36% of variance in clinical insomnia according to the Nagelkerke R^2^ value. This figure clearly exceeds the amount of combined variance explained by the previous three models. A one-point increase on the Patient health questionnaire (a measure of individual’s depression symptoms, ranging from 0 to 27) corresponds to a 22% higher likelihood of experiencing clinical insomnia.

## Discussion

The presented results illustrate a clear and consistent effect of measurement level when considering the effect of digital technologies on insomnia. Variables, measuring addictive use of technology, are the most effective predictors of both insomnia symptoms and clinical insomnia (around 10% of explained variance), while measures of technology use duration are much less informative, though still relevant (around 4% of variance explained). Meanwhile, the most basic measurement level, i.e. whether a certain technology is used, yields an almost negligible effect on insomnia (around 0.5% of variance explained).

Our findings also indicate an important effect of digital technology type. As a rule, measures of social media use consistently outperformed measures of internet use and video game use in terms of their ability to predict insomnia. Interestingly, just being a social media user can be considered a minor risk factor for both insomnia symptoms and clinical insomnia. Thus, avoiding negative effects of social media on sleep may not be trivial even for users who do not use social media excessively or addictively. The detrimental effects of social media use on sleep quality in youth (16–25 years) have been previously documented in a systematic review by Alonzo and colleagues^34^. Conversely, measures of gaming exhibit weak or inconsistent associations with insomnia; information about whether someone is a gamer or how much time they spend gaming does not tell us a lot about their insomnia symptoms. A few obvious differences between social media and video games may be important in this context; scrolling through content displayed on any social media platform is likely less cognitively demanding than playing video games and is thus not difficult to do even in a sleep-deprived state; compared to gaming, it can also be performed more easily while lying in bed. Lastly, while video games commonly contain natural stopping cues (e.g., completed quest or challenge, end of game or match), social media platforms typically lack such cues (e.g. endless scrolling, auto-play feature).

In order to put the effect of our technology measures on insomnia into perspective, one measure of depression symptoms was included as a predictor in the model. Insomnia is known to have a high comorbidity with depression, and common causalities have been established between the two conditions^35^. In line with our expectations, one measure of depression symptoms explained significantly more variance in insomnia than all digital technology measures combined.

Furthermore, after taking depression scores into account, the effect of addictive use of internet and social media on insomnia decreased to a large degree. Two causal mechanisms may explain this finding; one possibility is that individuals who develop addictive use of internet and social media are more likely to start experiencing depressive feelings, which lead to sleep problems. However, after briefly consulting relevant literature, it seems that *social media use—sleep problems—mental health risk* is the more commonly proposed causal pathway. Alonzo and colleagues^34^ describe six studies proposing the latter pathway and only one study examining the former^36^.

A second explanation of our findings, i.e. the effect of social media and internet use on insomnia being explained by depression symptoms, argues that depressed users of internet and social media are much more likely to use these technologies as a way to alleviate depressive feelings. Since using social media for mood modification is considered a criterion for social media addiction^37^, it is unsurprising that addicted social media users are more likely to suffer from depression. Due to the seemingly large association between these two variables, it seems plausible that the effect of social media addiction (as well as internet addiction) on insomnia is primarily related to higher probability of experiencing depression symptoms.

These mechanisms could also apply for gaming, but to a lesser extent; addictive use of gaming holds the largest independent effect on insomnia among the three technology types. This implies that addictive use of video games does not only predict insomnia due to addicted gamers being more depressed, but also through other mechanisms.

### Limitations

Considering the notable differences in findings among various digital technology measures, it would have been desirable to study their effects on more measures of insomnia (e.g. using different questionnaires, as well as less subjective insomnia measures) or on other measures of sleep quality and quantity. Secondly, while hierarchical regression analysis of cross-sectional data is used for some speculation regarding causal pathways in this paper, it is far from proof of such relations between the phenomena. While it is not impossible that sleep problems lead to more digital technology usage, we expected that this direction of effect would account for only a minor proportion of the correlation between the two phenomena, compared to the (proposed) effect of digital technology use on sleep. Experimental and longitudinal research designs would have more merit in establishing conclusions of causal pathways between digital technology use and sleep.

## Conclusions

The effect of using digital technologies on the quality and quantity of our sleep is far from straightforward. The present research shows that this effect almost entirely depends on how we measure digital technology use. In terms of measurement level, it is apparent that more complex measures (e.g. addictive use of a certain digital technology) consistently outperform simplistic measures (e.g. duration of use) when it comes to predicting insomnia. In terms of technology type, social media use seems to produce more risk for insomnia, compared to playing video games or general internet use for leisure purposes, regardless of the measurement level. Interestingly, most of the effect of social media use on insomnia may be explained by understanding users' depression symptoms.

Successfully improving patients’ sleep may therefore require a focus on improving their self-control over technology use, rather than simply reducing their screen time. Efforts aimed at spreading awareness related to insomnia will benefit from including specific advice regarding (avoiding) social media use before sleep, and from illustrating the interrelationship between digital technology use and mental health. Our findings also form the basis for more specific research into how each of the various types of digital content may affect our sleep, in order to combat and eventually overcome the evident epidemic of sleep disorders.

## Data Availability

The datasets generated during and/or analysed during the current study are available from the corresponding author on reasonable request.
